# Preparation and Property Evaluation of Conductive Hydrogel Using Poly (Vinyl Alcohol)/Polyethylene Glycol/Graphene Oxide for Human Electrocardiogram Acquisition

**DOI:** 10.3390/polym9070259

**Published:** 2017-06-30

**Authors:** Xueliang Xiao, Guanzheng Wu, Hongtao Zhou, Kun Qian, Jinlian Hu

**Affiliations:** 1 Key Laboratory of Eco-Textiles, Ministry of Education, Jiangnan University, Wuxi 214122, China; xiao_xueliang@jiangnan.edu.cn (X.X.); 18084721450@163.com (G.W.); ycfyzht@163.com (H.Z.); qiankun_8@163.com (K.Q.); 2 Institute of Textiles and Clothing, The Hong Kong Polytechnic University, Hong Kong, China

**Keywords:** cyclic freezing-thawing method, conductive polymeric hydrogel, mechanical performance, electrocardiogram signal

## Abstract

Conductive hydrogel combined with Ag/AgCl electrode is widely used in the acquisition of bio-signals. However, the high adhesiveness of current commercial hydrogel causes human skin allergies and pruritus easily after wearing hydrogel for electrodes for a long time. In this paper, a novel conductive hydrogel with good mechanical and conductive performance was prepared using polyvinyl alcohol (PVA), polyethylene glycol (PEG), and graphene oxide (GO) nanoparticles. A cyclic freezing–thawing method was employed under processing conditions of −40 °C (8 h) and 20 °C (4 h) separately for three cycles in sequence until a strong conductive hydrogel, namely, PVA/PEG/GO gel, was obtained. Characterization (Fourier transform infrared spectroscopy, nuclear magnetic resonance, scanning electron microscopy) results indicated that the assembled hydrogel was successfully prepared with a three-dimensional network structure and, thereafter, the high strength and elasticity due to the complete polymeric net formed by dense hydrogen bonds in the freezing process. The as-made PVA/PEG/GO hydrogel was then composited with nonwoven fabric for electrocardiogram (ECG) electrodes. The ECG acquisition data indicated that the prepared hydrogel has good electro-conductivity and can obtain stable ECG signals for humans in a static state and in motion (with a small amount of drift). A comparison of results indicated that the prepared PVA/PEG/GO gel obtained the same quality of ECG signals with commercial conductive gel with fewer cases of allergies and pruritus in volunteer after six hours of wear.

## 1. Introduction

Conductive hydrogel, a soft adhesive combined with an Ag/AgCl electrode that can transfer human bio-potential from ions to electrons effectively, is widely used in bio-signal monitoring, for example, to monitor electrocardiograms (ECG) for patients with cardiovascular disease in hospital [1,2] or electromyogram (EMG) for human in fitness exercise [3,4] in real-time. This kind of hydrogel is required to attach to human skin tightly with high shape stability to avoid changes of the interface resistance and motion artifacts. However, the current conductive hydrogel in commercial electrodes manifests high adhesiveness to human skin. This adhesiveness of the hydrogel leads to an airtight layer to the skin, and such soft matter may cause human skin allergies and pruritus risk under long-term wear [5]. This is ascribed to the large amount of fragmented polymeric molecules in the gel [6], causing poor mechanical strength in the form of the adhesive and causing a prickling feeling in the skin leading to discomfort. Moreover, the poor mechanical performance of the hydrogel easily cause motion artifacts due to poor shape stability when applied to bio-signal monitoring. This requires new types of conductive hydrogel to be developed for high strength without an airtight layer when attached to skin, and good wear comfort, effective conductivity, and real-time and long-time bio-signal monitoring.

A hybrid hydrogel using conductive nanocomposites [7,8] may be a good candidate for ECG and EMG electrodes because of the reported highly-improved mechanical strength and conductivity. Hybrid hydrogel was reported with more than 80 wt % of water content [9]. The involved nanoparticles in the hydrogel still have a nanoscale effect and interface effect [10] that benefits the generation of large-scale physical crosslinking. Moreover, the hybrid hydrogel at the microscale was reported to have a unique three-dimensional network structure that can lock many types of functional elements inside, which shows broad potential applications in areas of biomedical engineering, such as drug carriers, anti-bacterial wound dressing, human cartilage, skin substitute material, medical gel electrodes, etc. [10,11,12,13].

In preparation of a biocompatible hydrogel, polyvinyl alcohol (PVA) has irreplaceable advantages, such as low cost, low toxicity, biocompatibility, and excellent chemical stability. Therefore, PVA is widely used as a key component in many biomedical materials [14,15]. Due to a large amount of hydroxyl groups on the PVA molecular chain, such a polymer has good water solubility and easily forms hydrogen bonds with other polar groups of molecules. It was reported that the cyclic freezing-thawing method is an effective method to construct a polymeric network with hydrogen bonds as crosslinkers [16,17]. A lower freezing temperature would lead to more and denser hydrogen bonding for higher strength of the produced hydrogel.

However, experimentally, it was found the pure PVA hydrogel had relatively poor mechanical performance after the cyclic freezing–thawing process, which may be ascribed to the limited number of crosslinkers in constructing the polymeric network that was formed by PVA macromolecule chains [18]. This leads to the restricted application of PVA gel. Thus, PVA usually requires compositing with another macromolecule polymer for formulating better mechanical performance [19]. For example, PVA hybrid composited with polyethylene glycol (PEG), which has a chemical formula of HO–(CH_2_–CH_2_–O)*_n_*–H shows evident existence of polar groups during the formation of crosslinking hydrogen bonds. In fact, PEG shows many unique natural features of a would-be biomaterial, such as being non-toxic, non-irritating and immunogenic, having good biocompatibility and biodegradability, and being soluble in water and many organic solvents (ethanol, acetone, etc.) [20,21]. In addition, from the view of processing, PEG has features of excellent lubrication, moisturizing, dispersion, bonding, and antistatic electricity properties. This indicates that such a polymer is a good candidate material for use in the pharmaceutical and bioengineering fields [22]. Similarly with PVA, pure PEG gel manifests relatively poor mechanical strength when water absorption swelling takes place. It is desirable to construct a polymeric network in a gel format using such a rich content of hydroxyl groups of PVA grafted with PEG molecules for developing biocompatible materials with high application value [23].

In addition, a hybrid composite of nanoparticles or complex ions (such as Fe^3+^ ions) into the hydrogel polymeric network is also an effective way to enhance the mechanical and swelling properties of hydrogel and endow the gel with some specific functions [24]. Herein, graphene oxide (GO) is a recently-popular research nanoparticle with good hydrophilicity due to the existing polar groups and somewhat conductive function in comparison with the higher conductivity of its origin, i.e., graphene [25]. The involvement of GO into polymer composites can improve the polymeric mechanical performance significantly due to the formation of crosslinking between GO polar groups and polymeric branches [26]. This is more like the complexation of heavy metal ions in polymer, such as the chrome tanning process of leather. On the other hand, GO has a large specific surface area that may benefit electron mobility compared with ion transfer in hydrogel porous channels. This can decrease the electro-resistance of the hydrogel when it contains GO distributed evenly across the gel [27]. GO is also a biocompatible nanomaterial and has been reportedly widely used in some biomaterial devices [28]. This paper introduces a self-developed hydrogel with high mechanical strength and conductivity using raw materials of PVA, PEG, and GO through the cyclic freezing-thawing method [29,30]. A number of characterization approaches are employed to prove the foundation of the hydrogel polymeric network among materials. The relationship of the mechanical performance and conductivity is carried out to indicate the self-developed hydrogel in bio-signal acquisition and wearable comfort. A demonstration of the developed hydrogel electrode is also conducted for its real application in ECG monitoring and accompanying biocompatibility.

## 2. Materials and Methods

### 2.1. Materials

Polyvinyl alcohol-1799 (PVA-1799) and graphene oxide (GO, average diameter 50–200 nm) nanoparticles were purchased from Shanghai Vita Chemical Reagent Co. Ltd., Shanghai, China. Polyethylene glycol 6000 (PEG-6000) was purchased from National Medicine Group Chemical Reagent Co. Ltd., Shanghai, China. Self-made circular polyester/cotton (65/35) acupunctured nonwoven fabric containing a male buckle in the center was manufactured for the substrate of the hydrogel electrode. Such fabric components showed a relatively stable shape in comparison with the typical sponge substrate.

### 2.2. Preparation of the Hydrogel and Bio-Signal Electrode

An explored process was specifically designed for preparing PVA/PEG/GO nanocomposite hydrogel, which may improve the mechanical properties and quality of the bio-signal in acquisition.

**Figure 1:**
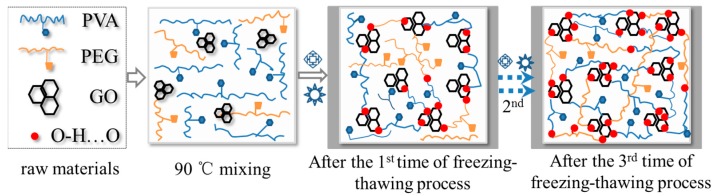
Schematic illustration of the formation mechanism of PVA/PEG/GO hydrogels using the freezing–thawing method (the mark 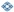 means freezing process, and 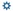 means thawing process).

(1) Raw polymers of PVA-1799 (4.00 g, –[CH_2_–CH(OH)]*_n_*–, average value of molecular weight is 1799) and PEG-6000 (4.00 g, HO–(CH_2_–CH_2_–O)*_n_*–H, average value of molecular weight is 6000) were blended into a flask with three necks, then 20.00 mL of deionized water was dropped into the flask;
(2) The solution was stirred at a rate of 600 r/min for 10 min at 90 °C, a transparent mixture of PVA-1799/PEG-6000 solution was then obtained;
(3) Stirring was continue at the rate of 600 r/min without heating; on the other hand, 0.30 g GO was dispersed into 10.00 mL deionized water under ultrasonic dispersion for 60 min;
(4) The dispersed GO solution was put into the stirring PVA-1799/PEG-6000 mixed solution, maintaining the stirring at a rate of 600 r/min for 30 min;
(5) The blended solution was poured into a PVDF pipe with an inner diameter of 10.00 mm for sample #1. The solution was then customed into an organic glass mold where a nonwoven fabric was in the mold as a substrate for sample #2. Then the pipe and mold were sealed with preservative films;
(6) The PVDF pipe and the organic glass mold were placed into a freezer which could supply a cold environment of −40 °C. The samples were maintained in such an environment for 8 h, then removed from the freezer for thawing for 4 h at room temperature;
(7) The freezing–thawing process was repeated three times for the generation of the PVA/PEG/GO nanocomposite hydrogel. An illustration for such a cyclic freezing-thawing method in preparing PVA/PEG/GO hydrogel is shown in Figure 1, where the raw materials of PEG and PVA were blended in hot water. After the addition of GO nanoparticles, the freezing and thawing processes were performed until the last image, which shows a large amount of crosslinking generated for high mechanical performance. Here, for the sake of comparison, the solution of step (2) can also experience the steps (5–7) for preparing PVA/PEG hydrogel. The hydrogel is then compared with the PVA/PEG/GO hydrogel in terms of mechanical properties and electrical conductivity.

In step (3), the content of GO nanoparticles in gel was constant. In fact, the GO content can be adjusted for investigating the different mechanical performances and conductivities. The other processing steps in preparing gel were the same. During the gel sample preparation, the devices used are listed as follows: 

A student power supply was used for the LED lighting demonstration (Jiangsu Tree Teaching Equipment Co., Ltd., Nanjing, China). A multimeter (VC890C^+^, Victory digital Co. Ltd., Shenzhen, China) was used for measuring the variation of the gel electro-resistance. An electronic balance (Mettler-Toledo Instrument (Shanghai) Co. Ltd., Shanghai, China) was employed for measuring the weight of the raw materials. A freezer (Aucma Co. Ltd., Qingdao, China) was employed for hydrogel preparation. A magnetic stirrer (DF-II, Changzhou Philip Experimental Instrument Co. Ltd., Changzhou, China) was used for stirring the blended solution. A high-power ultrasonic dispersion instrument (KH-400 KDB, Kunshan Hechuang Ultrasonic Instrument Co. Ltd., Kunshan, China) was similar with the stirrer for dispersing GO nanoparticles in solution. A vacuum freeze drier (LGJ-10, Beijing Songyuan Huaxing Co. Ltd., Beijing, China) was used for making characterization samples. A self-made organic glass mold and a cylindrical PVDF tube were used for making the hydrogel electrodes.

### 2.3. Hydrogel Characterization

The as-made PVA/PEG/GO hydrogels were processed using freezing and drying for dried samples (−80 °C and vacuum processing conditions until the samples were in solid and brittle states), a piece of a sample was cut by a laser blade and then gold-coated by sputtering for observation of the internal gel morphology under a scanning electron microscopy (SEM, JEOL-JSM-7500F, JEOL Co. Ltd., Tokyo, Japan, voltage 5 KV, current 20 μA). Here, during the freezing–drying process, the weight reduction of the hydrogel is assumed due to the escape of water molecules under the vacuum environment. An approximate calculation gives the porosity (∅) of as-made hydrogel based on Equation (1):

(1) $$\emptyset =\frac{w_{1}-w_{2}}{V_{1}\rho_{H_{2}O}}$$

where $w_{1}$ means the weight of wet hydrogel sample, $w_{2}$ means the weight of dried hydrogel sample, $V_{1}$ is the volume of wet hydrogel sample, and $\rho_{H_{2}O}$ is the density of water. The chemical component or the change of internal bonding of the milled dry hydrogel were characterized using a Fourier Transform infrared spectrometer (FTIR-5700, Thermo Nicolet Corporation, Beijing, China scanning range of 4000~400 cm^−1^, the resolution is 8 cm^−1^). The conventional KBr disk tablet method was chosen for the FTIR module. The NMR (nuclear magnetic resonance, MesoMR23-060V-I, Shanghai Niumag Co. Ltd, Shanghai, China) characterization of the PEG/PVA/GO gel was carried out using the medium of D_2_O. The variation or generation of the crystalline phase of the prepared hydrogels using the freezing-thawing process can be characterized by X-ray diffraction due to the Bragg regular arrangement of the crystalline phase. Thus, the crystallinity of the hydrogels from the regular gelation process, without generating crystals, and freezing-thawing process were determined and compared by a Rigaku Smart Lab XRD system (Regaku Co. Ltd, Tokyo, Japan, 9 KW) that is equipped with Cu Kα radiation with a wavelength of 1.54 Å. The hydrogels were cut in the format of a film to cover the glass stage. The test 2θ range is from 5° to 40° and recorded at a scan speed of 10°∙min^−1^ at 40 KV and 40 mA. The mechanical properties of as-made hydrogels were measured using an Intron-5566 machine (Instron Co. Ltd., Shanghai, China) for hydrogel breaking strength and strain where the hydrogel sample was standardized as a circular shape (0.8 cm in diameter and 10 cm in length). The relationships of the gels’ breaking strength and breaking elongation were plotted with the content variation of GO in the gels. Herein, the GO content of PVA/PEG/GO hydrogel was varied in the weight content range from 0.15 to 1.2%. At the same time, the bulk resistances of the cylindrical hydrogel under different stretched states were determined using a multimeter (VC890C^+^ type, Victory digital Co. Ltd., Shenzhen, China). The relationship of the tensile strain and measured resistance can be used for judging the linearity, sensitivity, and stability of as-made hydrogel conductivity. A qualitative demonstration of hydrogel conductivity was carried out using an LED lamp (Shenzhen Outai photoelectric Co. Ltd., Shenzhen, China).

A wearable belt for ECG acquisition was established, which integrated with a self-developed electro-circuit, a chip, Bluetooth, and four electrodes using the self-made hydrogel. The belt was stretchable, and the wearing tightness could be adjusted in terms of human chest circumference. The four electrode male buckles for mounting the hydrogel electrodes were placed according to ECG acquisition standards. The hydrogel electrode consists of a coin-shaped piece of hydrogel and a fabric substrate. Regarding the preparation of the ECG electrode, the as-made hydrogel was input on a nonwoven fabric and after experiencing the above-mentioned freezing–thawing conditions, the electrodes were then developed as disposable electrodes for ECG monitoring. One healthy volunteer without any cardiovascular diseases agreed to take up the ECG acquisition using the self-developed belt and hydrogel electrode. Here, in detail [31] for wearing such a belt, two electrodes were positioned on the human chest surface (marked as #l and #2) near the heart, while a third reference electrode (#3) was placed on the lower left position that is 10 cm away from electrode #1. This arrangement enables the production of relatively neat and clean (low noise) ECG signals with a higher R-peak amplitude and QRS complex waves. For a specific hydrogel-based electrode, its one fabric side was attached to human skin, while the other side was connected to the developed wearable belt through the metal buttons. The acquired signals can be visualized and stored to a specific base station. In order to check the quality of the electrodes in bio-signal monitoring, a comparison was carried out for the signal quality between static and dynamic states.

## 3. Results and Discussions

### 3.1. Synthesis and Morphology Analysis of the PVA/PEG/GO Hydrogel

**Figure 2:**
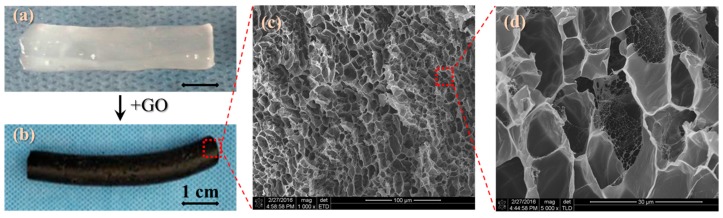
Hydrogel samples of (**a**) PVA/PEG; (**b**) PVA/PEG/GO; and SEM images from (**b**) that (**c**) shows the porous structure of gel under cooled and dried states under 1000× magnification; and (**d**) is a small area of (**c**) under 5000× magnification.

As shown in Figure 2a, the PVA/PEG gel is in a semi-transparent state, and is spread over a nonwoven fabric. The PVA/PEG gel was tested with a breaking strain of 120 ± 10% and a breaking strength of 20 ± 5 KPa, which were much lower than the mechanical performance of PVA/PEG hydrogel with GO nanoparticles. After involvement of GO nanoparticles into PVA/PEG sol, the composite hydrogel becomes black in a stable and highly-elastic state, as shown by the black cylindrical sample in Figure 2b. When the black hydrogel was cooled and dried with only the polymeric skeleton left, the morphology was captured clearly, as shown in Figure 2c at 1000× magnification. Evidently, the hydrogel skeleton is a porous medium that each pore, in the wet state, stores aqueous molecules. The cavity walls are rather thin and the internal pores are connected through narrowed “nozzle-shaped” throats, and smaller porous sheet nets are found inside each pore, forming porous walls that enhance the resistance of ions transfer, as shown in Figure 2d.

An approximate calculation indicates that the porosity of the as-prepared hydrogel is 90%, according to Equation (1), which means that the around 90% of the volume of hydrogel stores small polar molecules that can be an ion transition medium. However, the gel porous structure with complex ion channels may be the reason for the high electro-resistance of the as-made hydrogel. Herein, the cyclic freezing–thawing process is an effective way in constructing the hydrogel porous structure. The addition of GO nanoparticles into the hydrogel can enhance the structural stability and elasticity. The inherent black color of GO dominates the final hydrogel’s color. It is supposed that GO offers a stronger physical crosslinking platform because the GO surface consists of a large number of hydroxyl groups and ester groups that provide them the opportunity to be crosslinked with other polar groups from PEG and PVA macromolecular chains [26]. The freezing process at −40 °C can rapidly lock the crossover part of macromolecules and nanoparticles, the hydrogen bonds are thereafter generated and locked under such an extreme environment. Theoretically, the density of the formed hydrogen bonds at the macromolecule crossovers becomes thicker (possibly due to the formation of a crystalline phase) with the increase of repeated numbers of freezing–thawing process, as well as the decrease of low temperatures [32].

### 3.2. Characterization of Crosslinking Formation of the PVA/PEG/GO Hydrogel

**Figure 3:**
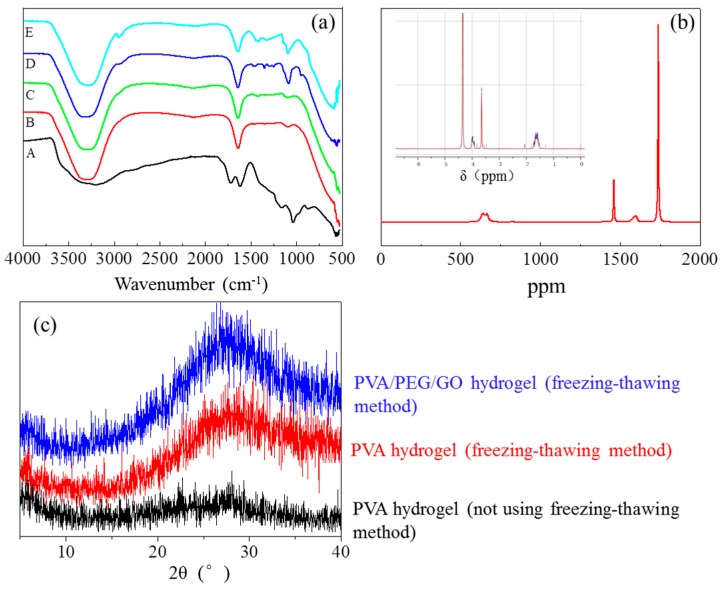
Crosslinking formation of hydrogel using characterizations of (**a**) infrared spectra of five materials, respectively, A—GO, B—PVA/PEG hydrogel, C—PVA, D—PEG, and E—PVA/PEG/GO hydrogel; (**b**) NMR spectra of PEG/PVA/GO hydrogel with a δ value from 1 to 2000 where the inset is a transition of the abscissa from ppm to δ; and (**c**) XRD raw data of the PVA hydrogel using and without using the freezing–thawing method, and PVA/PEG/GO hydrogel using this gelation method; the raw spectra are marked with related interpretations, respectively.

Figure 3a shows five infrared spectra of related materials to hydrogels by means of comparing their characteristic absorption bands, i.e., GO, PVA, PEG, PVA/PEG, and PVA/PEG/GO hydrogels. Among the five materials, PVA and PEG both have a large number of hydroxyl groups (–OH), thus, their IR spectra manifest similar characteristic peaks [29,30], such as 3280 cm^−1^, 1412 cm^−1^, and 1087 cm^−1^ of strong absorption peaks by O–H stretching vibration, CH–OH in-plane bending vibration, and C–O stretching vibration caused by the hydroxyl characteristic peaks respectively. The characteristic peaks at 2943, 1325 and 849 cm^−1^ represent the symmetric stretching vibration of C–H, in-plane bending vibration of C–H, and stretching vibration of C–C caused by the carbon chain characteristic peak, respectively. However, the PVA/PEG hydrogel’s infrared spectrum (Figure 3a(B)) shows some displacements of hydroxyl characteristic peaks from the original 3280, 1412 and 1087 to 3320, 1422 and 1092 cm^−1^, respectively [33]. Meanwhile, the peak intensities have increased to some extent, too, and there is also a twin-peak found at 1096~1110 cm^−1^, which means a formation of a grafting structure caused by PEG at both ends of the hydroxyl grafted onto PVA’s hydroxyl groups to form a crosslinking network. The conductive nanoparticles (GO), have a few IR characteristic absorption peaks, as shown in Figure 3a(A); that is, 3400 cm^−1^ for the strong and wide –OH stretching vibration peak, 1730 cm^−1^ for the corresponding COO^−^ of the C=O stretching vibration, 1421 cm^−1^ for the O–H deformation and vibration, and 1048 cm^−1^ for the C–O stretching vibration. 

Due to GO being at the nanoscale, the large surface area ratio leads to GO with a high opportunity in the generation of physical crosslinking. After the addition of GO into PVA/PEG hydrosol (GO content is 1.2 wt %), Figure 3a(E) shows the IR spectra of PVA/PEG/GO hydrogel, in which the formation of crosslinking presents evidently through the wavenumber shifting and peak intensity. Here, the characteristic peak of the stretching vibration of –OH group shows a displacement at 3280 from 3320 cm^−1^, the in-plane bending vibration of the peak of the –OH group is moved to 1430 from 1640 cm^−1^, and the stretching vibration of peak of C–O is moved to 1088 from 1098 cm^−1^. The lower wavenumber shifting of the characteristic peaks of key polar groups indicates a strong formation of hydrogen bonds between GO and PVA and PEG. This formation of physical crosslinking significantly benefits the hydrogel with high mechanical performance.


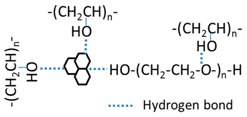
(2)

The NMR characterization of the PEG/PVA/GO gel shows the existence of –OH, –CH_2_–, and –CO–, etc. especially along the macromolecular chains for the branches of hydrogen H^+^, indicating a suggested structure of (2) formed between macromolecules of PEG and PVA and nanoparticles of GO. For example, the characteristic peaks of δ equaling 4 and 1.6 mean the existence of a hydrogen proton on –CH_2_–, and 3.6 and 4.3 mean the hydrogen protons of hydroxyl groups at different molecular chains. Especially for the ppm values equal to 1460 and 1750, the two characteristic peaks prove the existence of polar groups, such as –OH, –COOH, or –COOC– on the surface of GO nanoparticles that have been processed after oxidation treatment. The NMR spectra, in combination with the FTIR spectra of the PEG/PVA/GO gel from separate spectra of the components, infer that a large amount of hydrogen bonding as crosslinking segments have been generated in the final porous structural hydrogel.

To understand either generation or variation of the crystalline phase, we also examined the XRD patterns of the physically-crosslinked PVA and PVA/PEG/GO hydrogels, as shown in the raw data of the three patterns in Figure 3c. It is found that one strong and broad peak occurs at approximately 27.8° for PVA and PVA/PEG/GO hydrogels made from the freezing–thawing process. This indicates the dense hydrogen bonding as analyzed in Figure 3a as the form of physical crosslinking may assemble regularly into a crystalline phase under extreme low temperatures and cyclic transfer of freezing and thawing. In comparison with PVA, the hydrogel without the crystalline phase, using a regular gelatin process, such as ultraviolet-light gelation, and freezing process to hydrosol promotes the generation of the crystalline phase for the orientation of hydrogen bonds or the assembly of such crosslinkers in regularity. Furthermore, the slightly higher intensity of PVA/PEG/GO hydrogel indicates the greater amount of the crystalline phase generated because of the involvement of GO nanoparticles as the crystalline nucleus in the hydrogel system under freezing-thawing conditions. This is consistent with the resulting data of the IR spectra.

### 3.3. Analysis of Mechanical Properties

**Figure 4:**
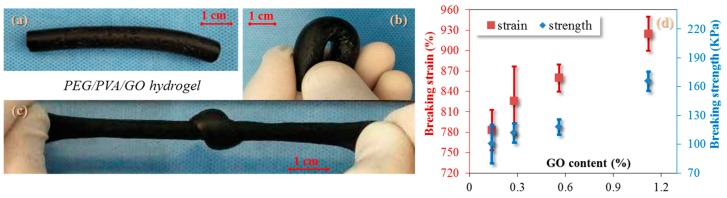
Mechanical analysis of conductive PVA/PEG/GO hydrogel in (**a**) the original state; (**b**) the bending state; (**c**) the knot-stretching state; and (**d**) the relationship of breaking strain and stretching with the increase of GO content.

Although the developed hydrogel is a porous medium, a good mechanical performance is noted for its stretching and elasticity. A comparison was performed for the cylindrical hydrogel in bending and stretching. Evidently, the hydrogel manifests as bendable with an appropriate modulus and high elasticity (at least three times the tensile strain of the original state (the original state of the sample in Figure 4a shows parameters of 8.60 mm in diameter and 60 mm in length)) under a knot tensile, as shown the deformation demos in Figure 4b,c. Quantitatively, a stretching process was carried out for testing the hydrogel with the parameters of breaking strain and strength under variety of GO contents. The results are shown in Figure 4d.

An increasing tendency is obviously noted for both parameters with the increase of GO content. The higher content of GO in the hydrogel shows a higher breaking stress, which may be ascribed to the formation of more crosslinks between GO nanoparticles and PEG/PVA macromolecules after the cyclic freezing process. The breaking strain reaches nearly 1000% when GO content (weight index) of the hydrogel is more than 1%. This indicates the high significance of GO in improving the mechanical performance of PEG/PVA hydrogel. A reference hydrogel of PEG/PVA gives only a 120% breaking strain, indicating the improvement of GO in the elastic strain for releasing more stretched molecule chains with the aid of GO. The high mechanical performance benefits the hydrogel in the ECG electrode application because such electrodes are not so attachable to human skin without an airtight layer on skin, which may avoid erythema or allergic reactions of human skin.

**Figure 5:**
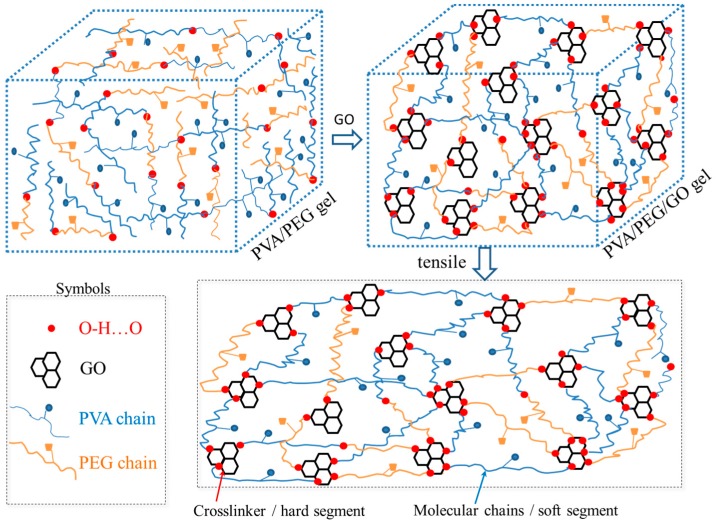
Schematic illustration of the molecule network of PVA/PEG/GO hydrogel from addition of GO nanoparticles into PVA/PEG sol before the freezing-thawing approach, and the deformation of the gel network under stretching, where the crosslinker on GOs means hard segments provide entropic elasticity and soft segments undertake the large tensile strain.

From the microscale view of hydrogel deformation in detail, the prepared PEG/PVA creates the polymeric network through pure hydrogen bonds among polar groups. The poor mechanical property may be ascribed to the small number of hydrogen bonds generated between connection points of two macromolecule backbones and branches. Varaprasad [34] suggested that GO has a strong ability to adsorb unrestrained macromolecule branches because of the high specific surface area of GO nanoparticles with high adsorption energy. Moreover, different from graphene, the surface of GO has a large number of polar groups (e.g., –OH group) during oxidation reactions, which provide a high chance in the generation of physical crosslinking (i.e., hydrogen bonds). As shown in Figure 5, the original state of the PEG/PVA/GO hydrogel illustrates a large number of connection points between PEG, PVA, and GO molecules, where GO nanoparticles offer the key positions for the generation of crosslinking.

A stretching process to the hydrogel sample imparts tensile strain several times. Like other elastomers (e.g., polyurethane) [35], the crosslinking points act as hard segments for maintaining the hydrogel shape, while the macromolecule chains between hard segments with conformational variations can store the stretched stress for subsequent strain recovery. The breaking point of the hydrogel under stretching usually takes place at the thinnest wall of the gel pores that have undertaken the largest stress concentration.

### 3.4. Electrical Conductivity of PVA/PEG/GO Hydrogel and Its Application in ECG Acquisition

#### 3.4.1. Conductivity of PVA/PEG/GO Hydrogels

**Figure 6:**
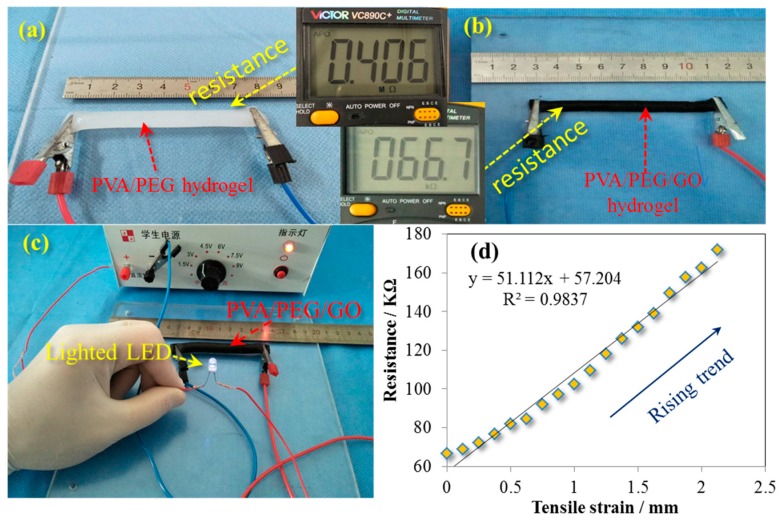
Conductive properties of (**a**) cylindriform PVA/PEG hydrogel; and (**b**) PVA/PEG/GO hydrogel; (**c**) a demo on lighting an LED lamp using PVA/PEG/GO hydrogel with such a high resistance; and (**d**) the relationship of gel electrical resistance and gel tensile strain under uniaxial stretching.

Apart from difference of mechanical performance, the as-made hydrogel has an evident difference for electro-conductivity with and without GO nanoparticles. Figure 6a,b compare the resistance of two hydrogels under the same length and cross-section shape and area, and find that PVA/PEG/GO gel has approximately one-sixth of the resistance value of the PVA/PEG gel. This indicates that the addition of GO nanoparticles into the PVA/PEG sol benefits the conductivity of the as-made gel. The reason may be that GO’s resistance is smaller than that of the PVA/PEG hydrogel. As shown in Figure 5, the involvement of GO nanoparticles may assemble a few electrical paths along the gel in a circuit. However, due to the oxidation of graphene (a highly-conductive material), GO becomes semi-conductive because of a grafted thin layer of polar groups on the graphene surface. The layer thickness or the conductivity of GO depends on the degree of oxidation. This may be the reason for the kilo-Ohm scale of the PVA/PEG/GO hydrogel electric resistance. The measured resistance of a cylindrical PEG/PVA/GO hydrogel (8.60 mm of diameter and 80 mm length) is 67 KΩ. However, a small LED bulb still can be lighted under 3 V using the hydrogel to connect the circuit, as shown in Figure 6c. This indicates the developed hydrogel can absolutely be an ECG electrode material, in comparison with the commercial ECG electrodes that have resistances on the scale of KΩ and MΩ (such as the Solar electrode with 2 KΩ and the 3M electrode with 0.8 MΩ resistances) [36]. 

Regarding the dynamic resistance of the as-developed hydrogel, a few values of static electro-resistances were recorded with the increase of hydrogel length. Herein, the original geometric factors of the PVA/PEG/GO hydrogel were 8.60 mm in diameter (cylindrical shape) and 80 mm in length. During the stretching of hydrogel, it was noted that the increase of resistance reveals a slight nonlinearity when the strain is increased up to 0.5 (from 80 to 120 mm), and the subsequent relationship of the hydrogel resistance and length manifests a linear trend, as shown the trendline with the fitted equation and *R*^2^ value (0.9837) for the tested scattered data in Figure 6d. This linearity accords with the intrinsic resistance (*R*) of a basically homogenous material that manifests a linear relationship of resistance and length:

(3) $$R=\frac{\rho L}{S}$$

where ρ is the resistivity, *L* is the length of gel, and *S* is the cross-section area of hydrogel. This indicates that the GO nanoparticles disperse in the hydrogel relatively evenly. Furthermore, the developed hydrogel with high elasticity is a good candidate as a strain sensor, which may have great potential in the areas of fitness clothing, robots, etc. Then, a cyclic tensile test shows the original length of the hydrogel becomes a mere 2% longer than the original value after one thousand tensile cycles (100% strain in stretching), indicating the high stability of the developed hydrogel’s mechanical performance, which is consistent with Figure 5 for the function of hard segments in maintaining the gel structural integrity.

#### 3.4.2. Hydrogel in Human ECG Signal Acquisition

**Figure 7:**
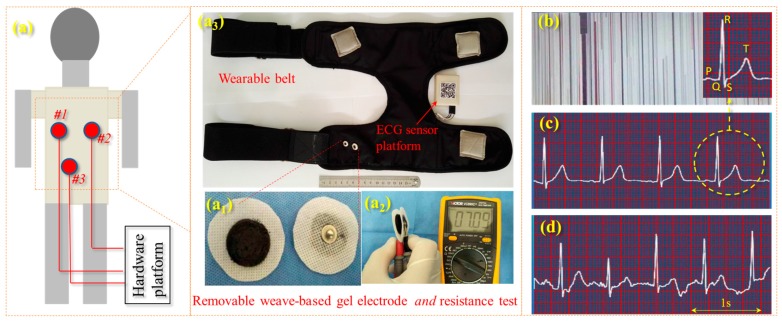
ECG acquisition process: (**a**) electrode distribution on the human body where (**a_1_**) shows our self-developed conductive hydrogel-based electrodes, (**a_2_**) shows the tested resistance of a pair of electrodes, and (**a_3_**) shows the self-developed ECG monitoring belt and integrated circuit for signal processing; (**b**) ECG noise when the belt does use the developed gel electrode, the inset shows a typical unit of ECG signal with characteristic waves of P, Q, R, S, and T respectively; (**c**) ECG signal for a human in a static state; and (**d**) the ECG signal for a human in a walking state. The scale bar represents 0.25 s per unit length for recording the heart rate.

As shown for the bio-signal electrode in Figure 7a_1_, the PEG/PVA/GO hydrogel is attached to a small circular non-woven fabric that has a male buckle in the center. The manufacture of such a composite electrode was through pouring the PEG/PVA/GO sol onto a nonwoven fabric surface opposite to the buckle in a self-made circular mold. After three cycles of the freezing-thawing process (−40 °C/8 h + 20 °C/4 h each time), it was found that the hydrogel was adhered to the nonwoven fabric tightly due to the high hydrophilic property of the fabric materials. The metal buckle attached the hydrogel tightly without air interface resistance, ensuring the smooth transition of bio-potential through the electrode medium. The resistance measurement of an electrode shows the resistance value of 3.545 KΩ, as shown in Figure 7a_2_, which is under the same order of magnitude of the commercial electrode resistance. Figure 7a_3_ shows our self-developed ECG monitoring belt that has four electrode mounting positions that can provide three-lead ECG signals. The belt has two pairs of elastic bands and Velcro, which can adjust the tightness for subjects wearing the belt. The ECG monitoring of a human was carried out using a standard Holter with a single lead, as shown in the three-point (one point in Figure 7a_3_ is not used) interaction of diagram in Figure 7a. The obtained lead is approximately oriented along the cardiac axis. This method is a method to produce high-quality ECG signals because the typical orientation of the cardiac axis is well-known in clinical options. Additionally, this method of standard placement has an intrinsic advantage in conveying useful information for inspection. In this study, the hydrogel electrode was used for measuring the ECG signals of a subject in static and walking states. 

Figure 7b shows the ECG noise of the subject without using electrodes, indicating the importance of electrodes in ECG monitoring. Figure 7c,d shows two screenshots of ECG-signal monitoring a subject in different states for the same person using the same electrodes that are mounted on the developed ECG acquisition system. Here, it can be judged easily that the self-developed electrode for people in a static state gives good and acceptable ECG signals, where the waves of P, T, and QRS are clearly visualized, indicating the self-developed wearable ECG system can be used for patients in monitoring their body conditions in the home environment. Compared with the quality of ECG signals, Figure 7d shows a slightly unstable status of the ECG signal, as the baseline drift takes place due to a slight motion artifact, making the diagnosis slightly difficult to recognize. However, the waves of R, T, S, and Q waves can also be identified; at least the information of the heart rate and T-wave can be obtained. To some extent, this can be used to evaluate the accumulation of some cardiovascular diseases.

**Figure 8:**
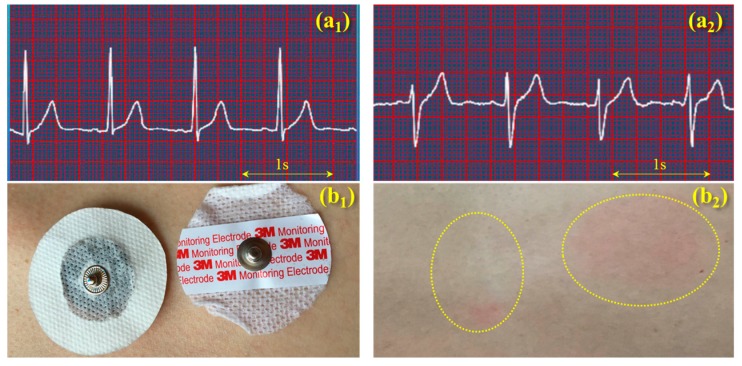
Comparison of the self-developed gel electrode and commercial 3M electrode in the performance of ECG acquisition and wearing comfort; (**a_1_**) ECG signal obtained from the self-developed gel electrode and (**a_2_**) the ECG signal from the 3M electrode, the scale bar means 1 s across four units for recording the heart rate; (**b_1_**) two electrodes attached on a human skin at the same time, and (**b_2_**) the skin reaction after six hours of wearing for the self-developed hydrogel (left) and the commercial hydrogel (right).

Figure 8 compares the performances of ECG monitoring and wearing comforts between the self-developed electrode (PVA/PEG/GO hydrogel with a metal buckle) and a commercial 3M electrode (conductive hydrogel with a Ag/AgCl buckle). The ECG signals were obtained from a volunteer in a static state (Figure 8a_1_,a_2_). The comparison of results shows both signals with high resolution, clear identification, and effective monitoring. A large amount of effective information of humans in normal conditions or chronic cardiovascular and cerebrovascular diseases can be obtained from both ECG signals, in terms of real-time heart rate, and features of QRS waves. This indicates that the two kinds of hydrogels have the same function in bio-signal acquisition and monitoring in comparison of their signal/noise ratio, wave amplititude, and characteristics. A comparison of wearable comfort was conducted between the two electrodes that attached to human skin, as shown in Figure 8b_1_, after six hours of adhesion, the self-developed electrode was still maintained in a hydrogel state, and it was found that the commercial electrode left a red circular area on skin (a kind of skin allergy) and the wearer reported a long time of uncomfortable prickling during the adhesion. On the contrary, the self-developed hydrogel left a much smaller red area after the adhesion. The wearer did not report any serious uncomfortable feeling during the adhesion. However, the reasons for the generation of the skin allergy and pruritus are still unknown and require further investigation. For the electrode using the self-developed conductive hydrogel, it should be noted that the hydrogel would become thin and stiff, gradully, after long-term (>one day) exposure in air, which indicates its disposable feature is the same as the commercial hydrogel electrode when applied to bio-signal monitoring.

## 4. Conclusions

In combination with silver and silver chloride, some commercial conductive hydrogels are usually used for human electrocardiogram (ECG) acquisition. This hydrogel was reported many times with human skin allergies and pruritus under long-term skin attachment. This paper introduces a new type of hydrogel for ECG acquisition, using polyvinyl alcohol (PVA), polyethylene glycol (PEG), and graphene oxide (GO). Such materials are all biocompatible, biodegradable, and have good hydrophilicity, making them good candidates for bio-signal acquisition, providing good mechanical properties and conductivity. After a cyclic freezing-thawing process, SEM images showed the prepared PEG/PVA/GO hydrogel with a high porosity and honeycomb-like porous structure. FTIR and NMR characterization results indicated hydrogen bonding generated between polar groups of macromolecules of PEG, PVA, and GO nanoparticles, providing crosslinking points for high mechanical performance. Stretch testing showed that the breaking strain can reach many times the original length, and the breaking stress can reach up to 180 KPa. It was found that the breaking strain and breaking stress are increased with the increase of GO content in the hydrogel.

Electro-conductive investigation was carried out, and it was found that GO can reduce the resistance of PEG/PVA hydrosol significantly. An important discovery showed an almost linear relationship between hydrogel tensile strain and its electric resistance, indicating the stable polymeric structure and good mechanical performance of the prepared hydrogel for ECG monitoring. The composite hydrogel ECG electrodes were mounted on our self-developed ECG wearable belt. The ECG acquisition from a volunteer showed clear and stable signals in a static state that can be used for medical ECG diagnosis. The ECG acquisition for people in a walking state manifested a slight drift of signal; however, this does not influence the judgment of the heart rate and main wave signals. The bio-signal acquisition measurement and allergy or comfortability test indicated that the prepared hydrogel is a good candidate for skin attachment for more than six hours of wear due to the improved mechanical performance and the decreased possibility for an uncomfortable feel on human skin.
